# Monocyte behaviour and tissue transglutaminase expression during experimental autoimmune encephalomyelitis in transgenic CX3CR1^gfp/gfp^ mice

**DOI:** 10.1007/s00726-016-2359-0

**Published:** 2016-11-09

**Authors:** Navina L. Chrobok, Alexandre Jaouen, Keith K. Fenrich, John G. J. M. Bol, Micha M. M. Wilhelmus, Benjamin Drukarch, Franck Debarbieux, Anne-Marie van Dam

**Affiliations:** 1 Department of Anatomy and Neurosciences, Amsterdam Neuroscience, VU University Medical Center, De Boelelaan 1118, 1081 HV Amsterdam, The Netherlands; 2 European Research Center for Medical Imaging, Developmental Biology Institute of Marseille, Aix Marseille Université, Campus de la Timone, Marseille, France; 3 Faculty of Rehabilitation Medicine, University of Alberta, Edmonton, Canada

**Keywords:** Cell crawling, Intravital microscopy, Immunohistochemistry, Multiple sclerosis

## Abstract

Leukocyte infiltration into the central nervous system (CNS) is a key pathological feature in multiple sclerosis (MS) and the MS animal model experimental autoimmune encephalomyelitis (EAE). Recently, preventing leukocyte influx into the CNS of MS patients is the main target of MS therapies and insight into cell behaviour in the circulation is needed for further elucidation of such therapies. In this study, we aimed at in vivo visualization of monocytes in a time-dependent manner during EAE. Using intravital two-photon microscopy (IVM), we imaged CX3CR1^gfp/gfp^ mice during EAE, visualizing CX3CR1-GFP^+^ monocytes and their dynamics in the spinal cord vasculature. Our observations showed that intraluminal crawling of CX3CR1-GFP^+^ monocytes increased even before the clinical onset of EAE due to immunization of the animals. Furthermore, intraluminal crawling remained elevated during ongoing clinical disease. Besides, the displacement of these cells was larger during the peak of EAE compared to the control animals. In addition, we showed that the enzyme tissue transglutaminase (TG2), which is present in CNS-infiltrated cells in MS patients, is likewise found in CX3CR1-GFP^+^ monocytes in the spinal cord lesions and at the luminal side of the vasculature during EAE. It might thereby contribute to adhesion and crawling of monocytes, facilitating extravasation into the CNS. Thus, we put forward that interference with monocyte adhesion, by e.g. inhibition of TG2, should be applied at a very early stage of EAE and possibly MS, to effectively combat subsequent pathology.

**Electronic supplementary material:**

The online version of this article (doi:10.1007/s00726-016-2359-0) contains supplementary material, which is available to authorized users.

## Introduction

Multiple sclerosis (MS) is a chronic inflammatory and demyelinating disease of the central nervous system (CNS) that manifests in a wide range of clinical symptoms including sensory, motor and cognitive disabilities (Bobholz and Rao 2003; Sospedra and Martin 2005). About 85% of MS patients show a relapsing–remitting disease course with a pathological inflammatory phenotype characterized by massive influx of leukocytes from the circulation into the CNS (Kornek and Lassmann 2003). This cellular influx results in the local production of inflammatory mediators, activation of resident glial cells, demyelination, oligodendrocyte cell death and eventually axonal damage (Kornek and Lassmann 2003). So far, preventing the influx of leukocytes is the main target of recently developed MS therapies. For instance, the drug natalizumab blocks α_4_β_1_-integrin and thereby largely prevents interaction of leukocytes with the endothelium (Pucci et al. 2011).

Of the infiltrating leukocytes in MS, monocytes were shown to be essential for the development of clinical symptoms as investigated in experimental autoimmune encephalomyelitis (EAE), a well-known animal model of MS (Huitinga et al. 1990; Ajami et al. 2011). Infiltration into the CNS requires an orchestrated collaboration of chemokines and adhesion molecules from the inflamed endothelium and their receptors on monocytes. Monocytes in the bloodstream first roll on the activated and inflamed endothelial lumen, before they strengthen their adherence and crawl along the lumen to find an extravasation site into the inflamed CNS or detach and continue their journey in the vasculature (Ley et al. 2007; Man et al. 2007; Gerhardt and Ley 2015).

The multifunctional enzyme tissue transglutaminase (TG2) has been described to be involved in cell apoptosis and monocyte and macrophage adhesion and migration in vitro on fibronectin, a constituent of the extracellular matrix (ECM) (Akimov and Belkin 2001; Pankov and Yamada 2002). TG2 is upregulated under inflammatory conditions and was shown to be present in monocytes in active CNS lesions during MS and EAE (Iismaa et al. 2009; van Strien et al. 2015). Monocyte attachment to the endothelium and also differentiation into macrophages lead to a boost in TG2 expression (Metha et al. 1987; Eckert et al. 2014). TG2 present on the surface of monocytes and macrophage can act as a co-receptor for β_1_- and β_3_-integrins, which are crucial for the adhesion of monocytes (Akimov and Belkin 2001; Laudanna et al. 2002). This interaction with integrins enhances the affinity of the cells to extracellular matrix proteins, e.g. fibronectin, and therefore promotes monocyte adhesion and migration (Akimov and Belkin 2001). Recent studies from our and other groups have shown that knockdown of TG2 or pharmacological inhibition of its activity in rodents suffering from EAE led to reduced disease symptoms (Oh et al. 2012; van Strien et al. 2015). This could, at least partly, be explained by limited monocyte migration into the CNS (van Strien et al. 2015), but no detailed information about cellular behaviour in vivo in this context is known yet.

Nowadays, the presence of demyelinating lesions in MS animal models can be visualized in vivo by magnetic resonance imaging (MRI) (Rausch et al. 2003). Furthermore, intravenous injection of a contrast-enhancing agent reveals blood–brain barrier leakage in MRI, suggesting the presence of infiltrating cells but not providing information about the cell types being present (Rausch et al. 2003). Phagocytic cells in the lesions can be identified by MRI after injection of ultrasmall superparamagnetic iron oxide (USPIO) particles or USPIO-labelled antibodies specific for cell surface receptors (Pirko et al. 2003; Rausch et al. 2003; Floris et al. 2004). Although these techniques are non-invasive and provide relevant in vivo information on lesion formation, they lack detailed analysis and high resolution of specific cell types present in the spinal cord vasculature before and during clinical symptoms.

As we consider monocytes to be important players during the onset and development of clinical symptoms of MS and EAE (Kornek and Lassmann 2003), the aim of the present study is to investigate the interaction of monocytes with the spinal cord vasculature of EAE mice. For this purpose, we used transgenic CX3CR1^gfp/gfp^ mice which express green fluorescent protein (GFP) instead of the chemokine receptor CX3CR1. This genetic modification results in green-labelled monocytes and microglia cells. We imaged these CX3CR1-GFP^+^ cells with intravital two-photon microscopy (IVM), before and during symptomatic disease. Moreover, we analysed the presence of immunoreactive TG2 and caspase-3 in the spinal cord lesions previously analysed by IVM.

## Results

### Evaluation of EAE symptoms in CX3CR1^gfp/gfp^ mice with a spinal cord window

**Fig. 1:**
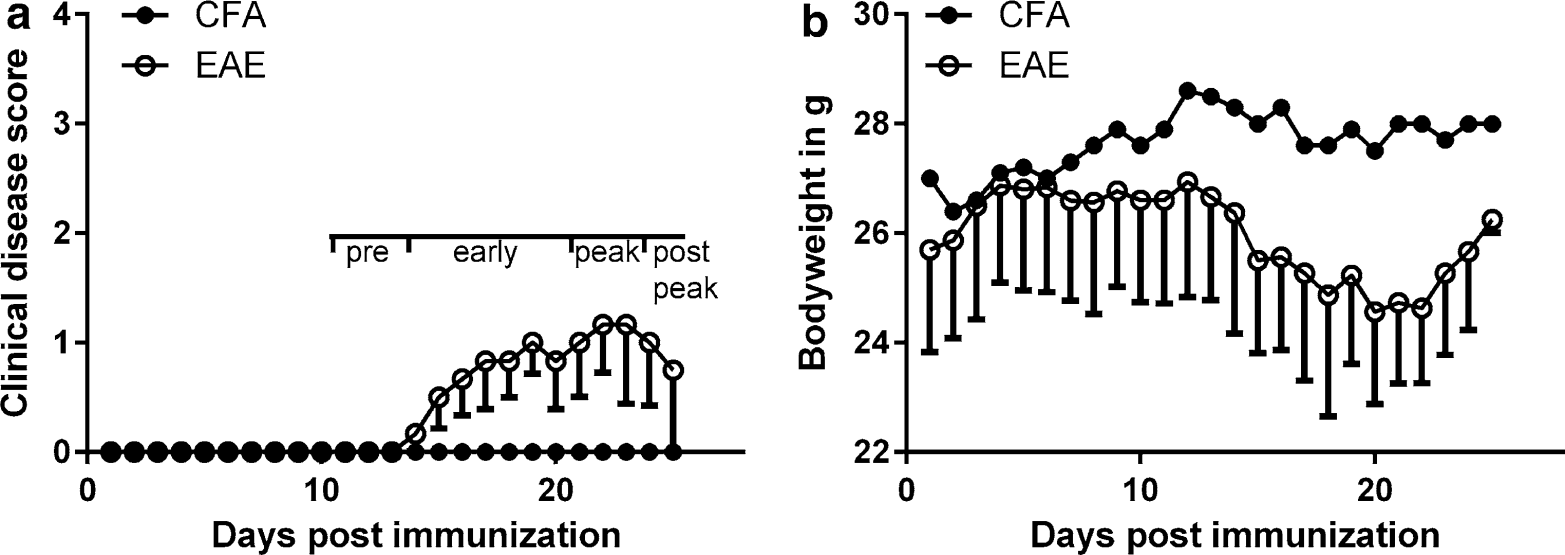
EAE in CX3CR1^gfp/gfp^ mice. **a** Clinical disease scores and **b** body weight after immunization with MOG_35-55_ (*unfilled circle*, *n* = 3) or CFA (*filled circle*, *n* = 1) in CX3CR1^gfp/gfp^ mice with spinal cord window implants. Data represent mean–SEM

The window-implanted CX3CR1^gfp/gfp^ mice developed a mild EAE disease course when immunized with myeloid oligodendrocyte glycoprotein peptide 35–55 (MOG_35-55_). The average maximal clinical score was around 1.5 (Fig. 1a) and the maximal loss of bodyweight was 8% (Fig. 1b). Naive and control animals (immunized with complete Freund’s adjuvant, CFA) did not exhibit clinical deficits or loss of body weight at any time during the experiment.

### IVM of the spinal cord parenchyma of CX3CR1^gfp/gfp^ mice

**Fig. 2:**
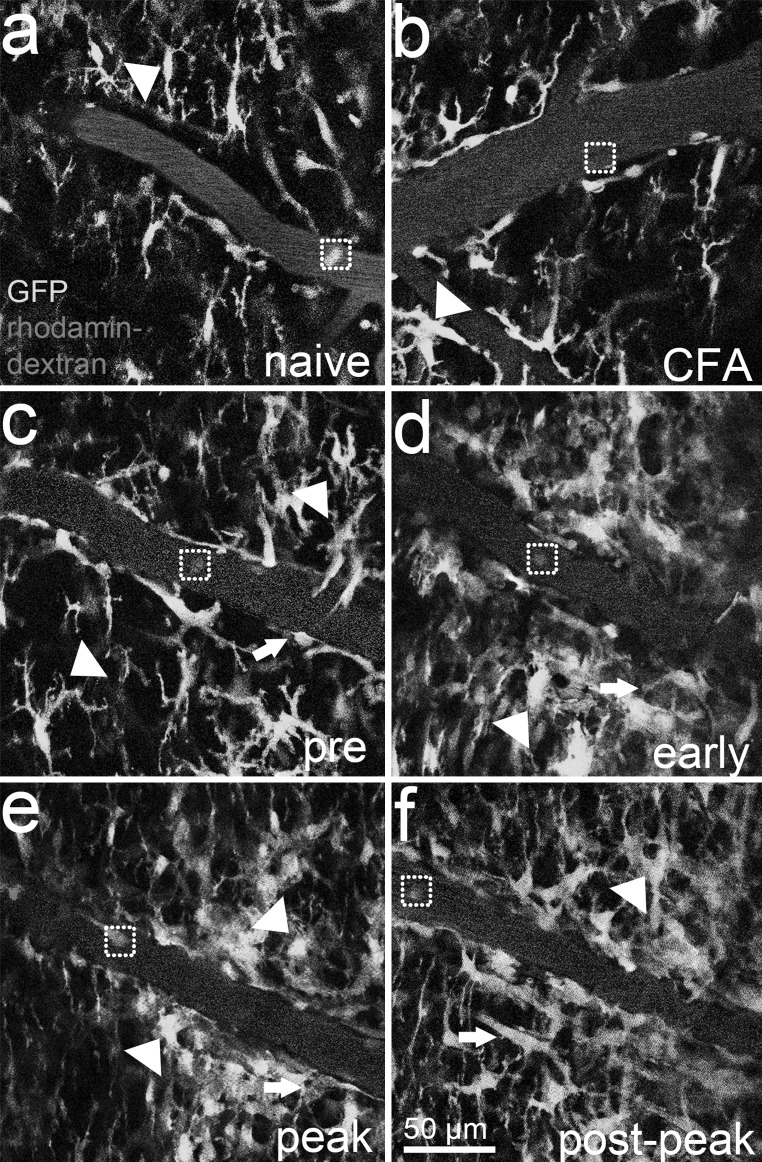
Representative two-photon IVM of CX3CR1-GFP^+^ cells in the spinal cord of CX3CR1^gfp/gfp^ mice. CX3CR1-GFP^+^ cells in the parenchyma (*arrowheads*) resemble microglial cells. They display a ramified phenotype in **a** naïve (*n* = 1) and **b** the CFA animal (*n* = 1). In MOG-induced EAE (*n* = 3) animals **(c**–**f)**, these cells morphologically change towards an amoeboid phenotype, with big cell clusters in symptomatic EAE **(d**–**f)**. Furthermore, during EAE **(c**–**f)**, the *red* Rhodamine B isothiocyanate-dextran (used to stain the blood vessels) leaks into the parenchyma where it is then taken up by cells, resulting in *red* cellular staining (*arrows*). In addition to parenchymal locations, **a**–**f** CX3CR1-GFP^+^ cells were observed within the bloodstream, specified by *squares* (colour figure online)

For in vivo imaging, we chose blood vessels draining into the central spinal cord vein on both the left and right side of the central vein in the region of the vertebrae T13 to L1 (corresponding to spinal cord levels L4 to S1, Harrison et al. 2013). Data from two-photon IVM (Fig. 2) revealed two distinct CX3CR1-GFP^+^ cell localizations in the spinal cord: in the parenchyma (arrowheads) and within the vasculature (squares). CX3CR1-GFP^+^ cells in the spinal cord tissue of the naïve animal (Fig. 2a) were microglial-like cells with a ramified morphology and thin processes, as previously shown (Jung et al. 2000). CFA immunization (Fig. 2b) seemed to bring the CX3CR1-GFP^+^ microglia at a closer distance to the blood vessels. After MOG immunization, the mice in the pre-symptomatic stage showed slightly more GFP signal in the parenchyma than the CFA animal (Fig. 2c). The parenchymal GFP signal further increased, especially in proximity to the blood vessel, coinciding with morphological changes of the CX3CR1-GFP^+^ cells towards a more amoeboid shape when the mice entered the symptomatic stages of EAE (Fig. 2d, e). During post-peak disease stage, still numerous amoeboid-shaped CX3CR1-GFP^+^ microglia were present (Fig. 2f). In EAE animals, also CX3CR1-GFP^+^ blood cells might have infiltrated the spinal cord, adding to the numerous CX3CR1-GFP^+^ amoeboid microglial cells, from which they cannot be distinguished, based on their morphology. Additionally, in the symptomatic stages of EAE (Fig. 2d–f), some red-coloured CX3CR1-GFP^+^ cells in the parenchyma close to the blood vessels were observed (arrows). This was likely caused by cellular uptake of Rhodamine B isothiocyanate-dextran leaked into the parenchyma. This was an EAE phenomenon and not detected in the naïve animal or the CFA animal (Fig. 2a, b) or in asymptomatic animals (data not shown).

### Cellular characterization of CX3CR1-GFP^+^ cells

**Fig. 3:**
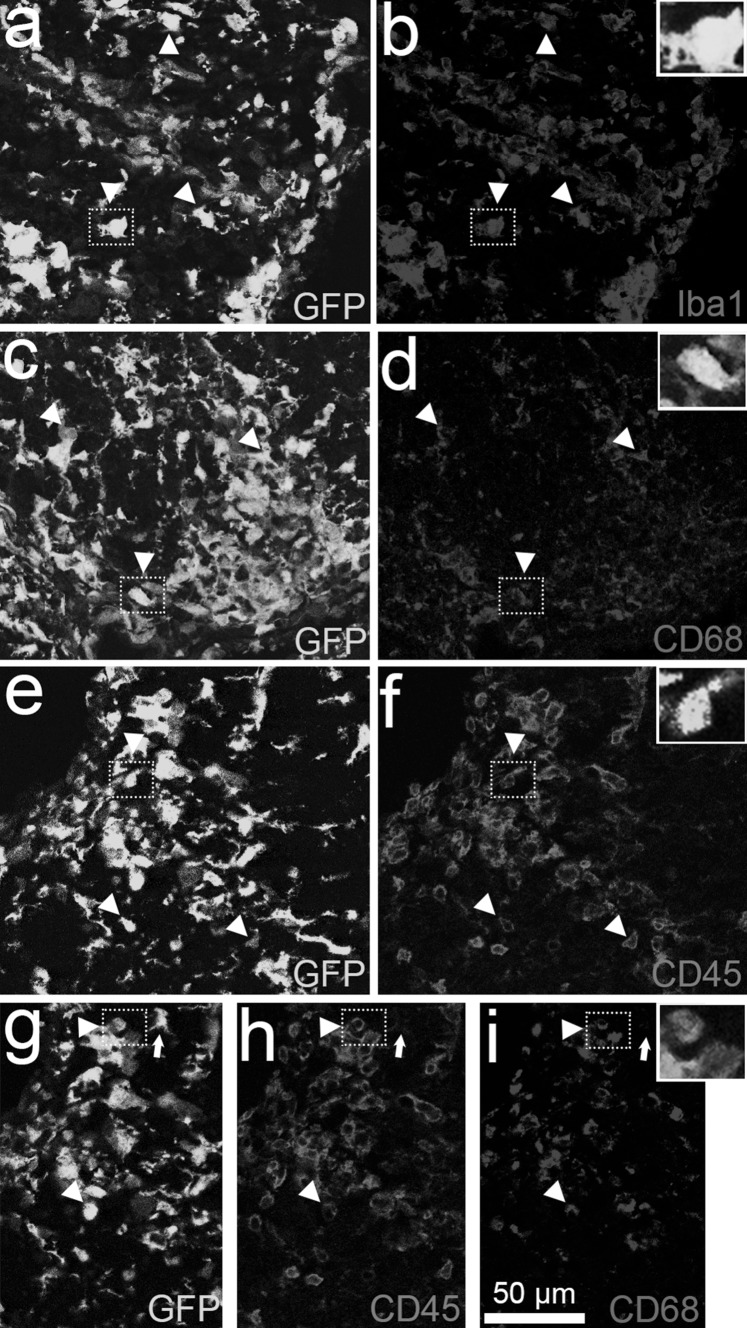
CX3CR1-GFP^+^ cells in EAE spinal cord tissue (*n* = 3) stained post-IVM are monocytes/macrophages and microglial cells. **a, c, e, g** Spinal cord EAE lesions in CX3CR1^gfp/gfp^ mice show clusters of ramified and amoeboid CX3CR1-GFP^+^ cells with immunoreactivity for **b** Iba1, **d** CD68 and **f** CD45 (*arrowheads*). Triple-positive cells for **g** GFP, **h** CD45 and **i** CD45 indicate that some GFP^+^ cells might have infiltrated from the bloodstream, while local ramified microglia (*arrows*) are GFP^+^ with low immunoreactivity for CD45 and none for CD68. *Insets* provide higher magnification of double-/triple-positive cells

**Fig. 4:**
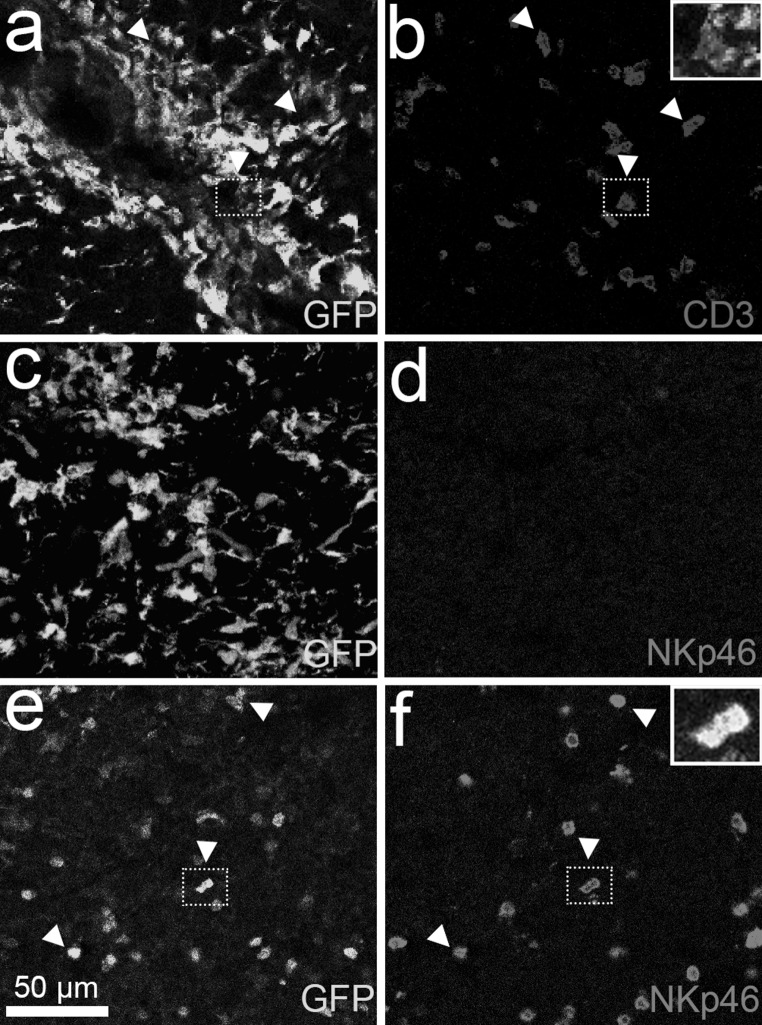
Neither T cells nor NK cells are amongst the CX3CR1-GFP^+^ cells in the EAE spinal cord tissue stained post-IVM (*n* = 3). **a**, **c** CX3CR1-GFP^+^ cells in spinal cord EAE lesions in CX3CR1^gfp/gfp^ mice do not display immunoreactivity for **(b)** CD3 (*arrowheads*, higher magnification in the inset) and **(d)** NKp46. **e, f** show GFP^+^ and NKp46 co-labelled cells in the spleen (*arrowheads*, higher magnification in the *inset*), which served as a positive control for the NKp46 antibody

To confirm the monocyte/microglia identity of the CX3CR1-GFP^+^ cells in the spinal cord of our CX3CR1^gfp/gfp^ mice induced with EAE, we immunohistochemically characterized these cells in the spinal cord area that had previously been imaged by IVM and are hence from post-peak disease (*n* = 3, Fig. 3). Our observations revealed that CX3CR1-GFP^+^ cells in the white matter lesions expressed markers for microglial cells and macrophages, i.e. Iba1 (Fig. 3a, b) and the phagocyte marker for lysosomal activity, CD68 (Fig. 3c, d). Furthermore, intense CD45 (Fig. 3e, f) immunoreactivity was found in the lesion areas, mainly in amoeboid-shaped cells (arrowheads and inserts). Moreover, co-localization of CD68 and CD45 in CX3CR1-GFP^+^ cells was found (Fig. 3g–i). We also observed ramified GFP^+^ cells that were weakly immunoreactive for CD45 but not CD68, indicative of local microglia (arrows). This finding supports the hypothesis of influx of CX3CR1-GFP^+^ from the vasculature into the spinal cord, although this was not observed during IVM. In a CFA animal, fewer and mostly ramified CX3CR1-GFP^+^ microglia were observed, uniformly distributed in the spinal cord (Supplement Fig. 1). These cells were all positive for Iba1 (Supplement Fig. 1a, b), but not for CD68 (Supplement Fig. 1c, d), indicative of a microglial phenotype. It has been described that CX3CR1-GFP^+^ cells can also comprise T and NK cells and therefore we stained for the presence of these cell types (Jung et al. 2000). CD3-positive T cells (Fig. 4a, b) appeared in the spinal cord lesions, although in low numbers compared to Iba1- and CD68-positive cells. Moreover, the CD3-positive T cells did not co-localize with GFP. NK cells were absent in the spinal cord lesions (Fig. 4c, d), although spleen tissue of the same animals showed NKp46^+^ cells that in small numbers co-localized with GFP^+^ cells (Fig. 4e, f).

### IVM of GFP^+^ cells in the vasculature

**Fig. 5:**
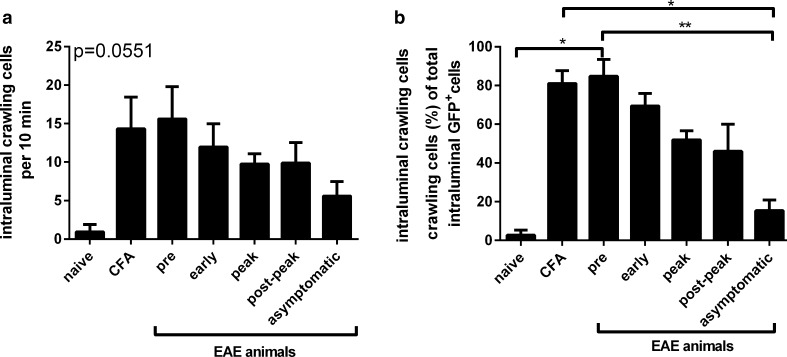
Quantification of the amount and percentage of intraluminal crawling CX3CR1-GFP^+^ monocytes during EAE. **a** The amount of crawling cells is expressed as the mean number of crawling cells counted per video in all available videos per disease stage or control and normalized to a time frame of 10 min +SEM; *p* = 0.0551, Kruskal–Wallis test. **b** The percentage of crawling cells of the total intraluminal CX3CR1-GFP^+^ cells increased significantly during the pre-clinical phase of EAE and remained elevated during on-going EAE compared to asymptomatic animals. The percentage of crawling cells per total number of intraluminal CX3CR1-GFP^+^ cells was calculated per video in all available videos per disease stage or control +SEM; *p < 0.05, **p < 0.01. The results presented include data from naïve (*n* = 1), CFA (*n* = 1), EAE (*n* = 2 per disease phase) and asymptomatic (*n* = 2) animals

Two-photon imaging revealed that CX3CR1-GFP^+^ cells in the vasculature of the naïve mouse rarely interacted with or crawled along the luminal side of the blood vessel endothelium (~1 cell per 10 min) as depicted in Fig. 5a. We termed these cells fast moving cells. An animal injected with CFA and subsequently imaged at a time point post-injection, comparable to the early disease phase in EAE animals, served as a control for the early stage of disease in EAE animals. The data showed a tendency of increased interaction of CX3CR1-GFP^+^ cells with the endothelium and around 15 cells per 10 min showed an extensive intraluminal crawling behaviour for longer than 25 s, which were designated crawling cells. A comparable amount of cells was observed in MOG-immunized animals during pre-symptomatic disease (Fig. 5a; pre). As EAE developed, the number of crawling cells was slightly reduced from pre-disease but remained elevated in all disease phases analysed, including post-peak disease. As another control, we imaged asymptomatic animals at a time point post-immunization that was comparable to post-peak disease. The number of crawling monocytes in asymptomatic mice was only half of the amount (~5 cells per 10 min) as found in post-peak EAE, indicating that the elevated crawling of monocytes is due to ongoing clinical EAE and not just due to the immunization as such.

A similar pattern of cell distribution was seen when the crawling cells were analysed as a percentage of total CX3CR1-GFP^+^ cells imaged within the circulation (Fig. 5b). CFA immunization, as well as immunization with MOG, resulted in a significant increase in the percentage of CX3CR1-GFP^+^ crawling cells within the mouse spinal cord vasculature of naïve to the pre-symptomatic EAE phase. The percentage of crawling cells progressively reduced significantly during the disease course from more than 80% in pre-symptomatic disease to 46% in the post-peak disease stage. In asymptomatic animals, only 15% of the cells were of a crawling phenotype, which was significantly different from the CFA-injected control animal and the pre-symptomatic EAE animals.

**Fig. 6:**
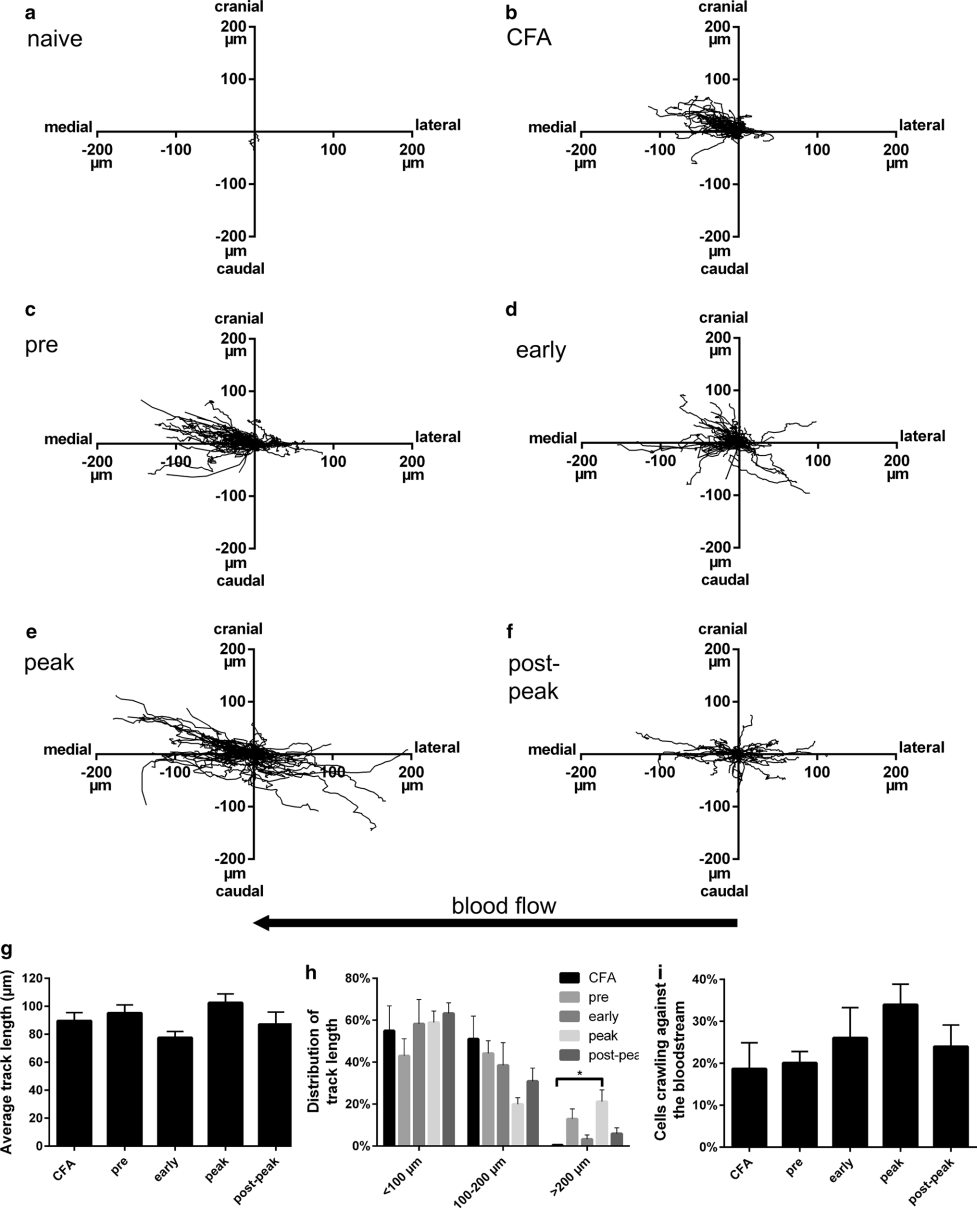
Tracks of intraluminal crawling CX3CR1-GFP^+^ cells. Monocyte crawling tracks are presented from **a** naïve (*n* = 1), **b** CFA (*n* = 1), and **c**–**f** EAE (*n* = 2) animals per disease phase. **g** Average track length of crawling cells. **h** Distribution of track lengths per animal group. **i** Percentage of crawling cells moving against the bloodstream. Coordinates of each cell track were set to the same origin to determine medial (in direction of blood flow) or lateral crawling of CX3CR1-GFP^+^ cells. Cranial and caudal direction of cell crawling depended on the orientation of the imaged blood vessel. All data are expressed as mean values +SEM

Additionally, we examined the crawling movement of monocytes during their interactions with the endothelium. Plotting the individual crawling tracks revealed that the displacement and the direction of crawling changed over time of the EAE disease course (Fig. 6). In the naive animal, as already shown in Fig. 5, only few crawling monocytes were present with a very limited local displacement (Fig. 6a). Consequently, they were excluded from further analysis. CFA immunization led to a displacement of monocytes up to about 100 µm (Fig. 6b). In EAE-induced animals (Fig. 6c–e), the displacement of crawling CX3CR1-GFP^+^ cells increased as disease progressed with a maximum at peak disease, displaying a displacement up to 200 µm. While the average track length presented little differences between CFA and EAE animals (Fig. 6g), the distribution of the track length was altered within the different EAE phases (Fig. 6h). Of all CX3CR1-GFP^+^ crawling cells, 50–60% displayed track lengths of less than 100 µm. Intermediate track lengths of 100–200 µm were similar during the different stages of EAE, although there was a tendency that they were reduced compared to the CFA-induced tracks (Kruskal–Wallis test, *p* < 0.05, post hoc test not significant). Long distance displacement of crawling cells extending 200 µm distance was only observed in MOG-immunized animals with a significant increase to around 20% of total crawling CX3CR1-GFP^+^ cells during peak disease. Furthermore, as already shown in Fig. 6b–f, CX3CR1-GFP^+^ cells did not only crawl in the medial direction with the bloodstream, but also in the lateral direction against the bloodstream (Fig. 6i). In CFA and pre-disease EAE animals, around 20% of CX3CR1-GFP^+^ crawling cells moved against the blood stream. With ongoing EAE, this cellular behaviour reached around 35% of CX3CR1-GFP^+^ crawling cells in peak disease.

### GFP^+^ cells in the spinal cord also express TG2

**Fig. 7:**
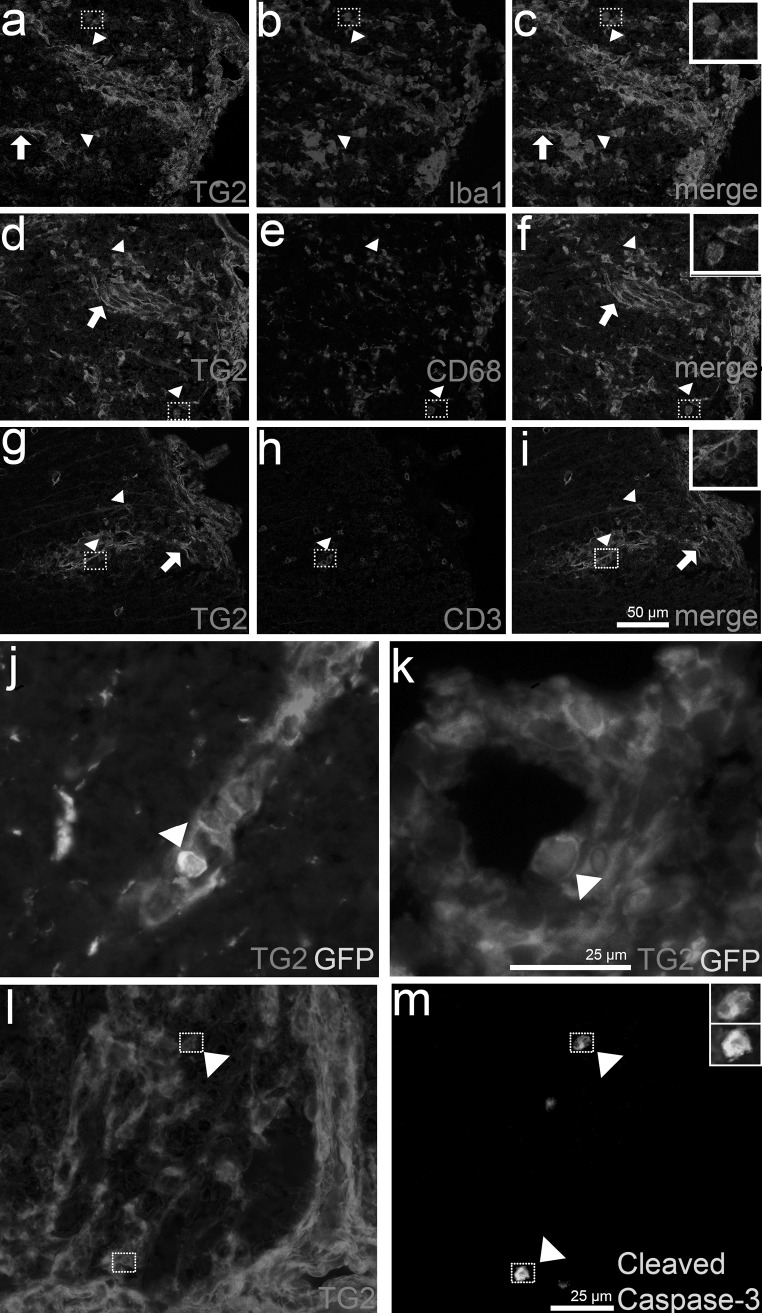
TG2 immunoreactivity in postmortem spinal cord of CX3CR1^gfp/gfp^ EAE mice (*n* = 3). **a**, **d**, **g** TG2 immunoreactivity is present in blood vessels (*arrows*) and cells (*arrowheads*) in spinal cord lesions of mice suffering from EAE. In these lesions **b** Iba1, **e** CD68 and **h** CD3 immunoreactivity is observed. TG2 is co-localized with **c** Iba1 and **d** CD68 and thus microglia and monocytes/macrophages, but not **i** CD3-positive T cells. **j, k** GFP^+^ and TG2 co-labelled monocytes can be found within the blood vessel (*arrowheads*). Blood vessels are presented in a sagittal **(j)** and coronal **(k)** direction. **l, m** Few cells in the EAE spinal cord lesions express cleaved caspase-3, which can co-label with TG2 (*arrowhead*). *Squares* represent cells shown at higher magnifications in the *insets*

To study whether TG2 is present in our mouse EAE model, immunohistochemical analysis of spinal cord sections was performed. TG2 immunoreactivity was found in the blood vessels of all EAE animals (Fig. 7a, d, g, arrows) and of the naïve and CFA control animals (as exemplified for the CFA animal in Supplementary Fig. 2), as described before (van Strien et al. 2015). Additional cellular TG2 immunoreactivity was found in spinal cord lesions of EAE animals.

GFP^+^ cells, identified as Iba1 and CD68-positive microglia and monocytes/macrophages, but not CD3-positive T cells in EAE lesions, showed TG2 immunoreactivity (Fig. 7a–i). Most TG2-expressing cells exhibited an amoeboid morphology which could be monocytes and monocyte-derived macrophages. Furthermore, ramified microglial cells in and near the lesion area were somewhat TG2 positive. Intriguingly, we observed TG2 immunoreactivity in CX3CR1-GFP^+^ cells within the blood vessel attached to the lumen (Fig. 7j, k). No extensive amounts of these cells were found due to perfusion of the animals, but particular cells attached to the endothelium were existent. As TG2 can contribute to immune cell apoptosis and is upregulated during this process (Sandor et al. 2016), we studied the presence of active caspase-3 in spinal cord EAE lesions. Only few cells immunopositive for the cleaved caspase-3 fragments (17/19 kDa) were found and single cells were co-labelled with TG2, while others were not (Fig. 7l, m). Overall, the presence of active caspase-3-positive cells in the EAE-affected spinal cord was limited.

## Discussion

The present study is one of the first to show the in vivo behaviour of circulating monocytes in the murine spinal cord at various time points after EAE induction. We observed an increased crawling behaviour of monocytes in CX3CR1-GFP^+^ transgenic mice in EAE- and CFA-immunized animals compared to a naïve control. This increase was most pronounced early after immunization and was observed in all animals that received adjuvants with or without MOG_35–55_. This indicates that this initial increase in monocyte crawling behaviour is rather due to general activation of the immune system, which is essential to induce EAE in MOG-immunized mice. Indeed, recent observations showed an increase in blood-derived monocytes already in the preclinical and early stage EAE (Barthelmes et al. 2016). We observed elevated intraluminal monocyte crawling during the early stages of EAE and, despite subsequent reduction to intermediate levels at later disease stages, the amount of crawling cells remained elevated in EAE mice compared to naïve and also asymptomatic animals. Therefore, an ongoing immune response as found in symptomatic EAE mice, which is absent in asymptomatic animals, seems needed to keep the crawling behaviour of monocytes elevated. Moreover, the displacement length of individual crawling CX3CR1-GFP^+^ monocytes was most extensive during peak disease. Furthermore, TG2-positive CX3CR1-GFP^+^ cells could be identified in and near the blood vessels during EAE, indicating a role of TG2 in mouse EAE in these animals.

To perform in vivo IVM analysis of monocyte behaviour, the spinal cord was made accessible by a window implantation. Although such a surgery could slightly delay EAE onset as described previously for the C57BL/6 strain, it does not alter the overall progression or disease score in this transgenic mouse strain (Fenrich et al. 2013a). The CX3CR1^gfp/gfp^ transgenic mice used in this study express GFP instead of the chemokine receptor CX3CR1 and are henceforth CX3CR1 knockout animals. While this receptor was shown to be involved in leukocyte adhesion and migration, the CX3CR1^gfp/gfp^ transgenic mice still develop EAE (Huang et al. 2006). Of importance, experiments comparing CX3CR1^gfp/gfp^ with heterozygous CX3CR1^+/gfp^ revealed that only the migratory properties of NK cells are affected during EAE in CX3CR1^gfp/gfp^ animals, leading to no NK cell recruitment to the EAE lesions (Huang et al. 2006). Although we cannot completely exclude changed monocyte crawling behaviour due to CX3CR1 depletion, the chemotaxis of monocytes and other leukocytes is not impaired as their recruitment to the site of inflammation is constant in homo-as well as heterozygous CX3CR1-GFP-expressing mice. We confirmed the impaired NK cell recruitment by the lack of NK cells in the spinal cord lesions, although NK cells expressing GFP were present in the spleen. For the first time, the described surgical approach of the spinal cord window implantation was used to access the spinal cord in the transgenic CX3CR1^gfp/gfp^ mouse strain. Our observation of CD68-, CD45- and/or Iba1-positive CX3CR1-GFP^+^ cells in the EAE spinal cord lesions, as well as the clearly developed clinical symptoms, supports the notion that these mice can be used for IVM to analyse monocyte behaviour during EAE.

Using IVM analysis, we found that CX3CR1-GFP^+^ are still able to crawl along the endothelial lumen, despite their loss of the CX3CR1 receptor and supporting previous data (Huang et al. 2006). Although the number of experimental animals used in this study was very small, some interesting changes in the behaviour of CX3CR1-GFP^+^ cells in the spinal cord vasculature were present between controls, EAE-induced asymptomatic animals and EAE-diseased animals and also within the different disease phases of EAE. Only few of the CX3CR1-GFP^+^ cells were found in the spinal cord vasculature of a naïve animal which corresponds to previous observations (Auffray et al. 2007; Carlin et al. 2013). When compared with the total number of intraluminal CX3CR1-GFP^+^ cells, the data suggest that under control conditions, most monocytes are fast moving cells that do not interact with the endothelium. However, upon treatment with CFA, more crawling monocytes were present at the luminal side of the blood vessel, likewise observed in the preclinical stage of EAE. This crawling monocyte behaviour remained present during the early stage of EAE and had a tendency to recede partially during continuing disease. Nevertheless, it seemed higher throughout disease compared with asymptomatic animals. Thus, activation of the immune system stimulates CX3CR1-GFP^+^ cells in the mouse spinal cord vasculature to enhance their intraluminal crawling behaviour, which remained present in EAE animals showing apparent disease symptoms. The observation that the percentage and number of monocytes interacting with the endothelium had a tendency to decrease as clinical disease progressed might be unexpected, but may reflect the steady increase in monocyte migration into the CNS during disease progression, as shown by pathological studies (Kuerten et al. 2007; Recks et al. 2011). Of interest is that a comparable disease-dependent behaviour has been shown for T cells in the same EAE model. This suggests that T cells and monocytes behave in a related time-dependent way during EAE (Kerfoot and Kubes 2002). Moreover, asymptomatic animals showed a low amount of crawling cells and also a low percentage of total CX3CR1-GFP^+^ cells in the vasculature, at a time point post-immunization comparable to post-peak disease. This is in line with the idea that the disease-induced change in monocyte intraluminal crawling behaviour contributes to or is a consequence of ongoing clinical EAE and not due to the immunization itself.

Subsequent analysis of the tracks of crawling monocytes showed that the average track length did not differ between the control and EAE animals, but there is a clear change in maximum displacement of the crawling cells as well as crawling direction over the disease course. In particular, during peak disease, numerous monocytes showed extended crawling displacement along the endothelium, not only in the direction of the blood flow but also against it. Both of these observations could be a consequence of firmer interaction with the endothelium mediated by soluble factors, e.g. chemokines and cytokines, secreted by local cells to actively attract more cells to the CNS (Redford et al. 1997; Leppert et al. 2001; Goldmann and Prinz 2013). Additionally, upregulation and activation of adhesion molecules on endothelial cells could be responsible for the extended crawling of monocytes in the lumen (Rossi et al. 2011).

Along with CX3CR1-GFP^+^ monocytes in the circulation, we observed CX3CR1-GFP^+^ cells in the spinal cord parenchyma. Under normal conditions, they reflect ramified microglial cells, but upon inflammatory conditions such as EAE, a clear increase in the amount of CX3CR1-GFP^+^ cells around the blood vessels becomes evident. It is, however, not possible to distinguish infiltrating monocytes from amoeboid microglia based on their GFP expression. Nonetheless, extensive immunoreactivity of the blood leukocyte marker CD45 on CX3CR1-GFP^+^ amoeboid-shaped cells in the spinal cord parenchyma indicates that indeed cellular infiltration of blood-derived CX3CR1-GFP^+^ cells had occurred. The density of ramified microglia around the blood vessels was increased since microglia migrate towards lesion sites, where the activated microglia can proliferate and enable phagocytising properties such as taking up myelin and cell debris (Yin et al. 2010; Ajami et al. 2011; Chastain et al. 2011). The phagocytic capacity of CX3CR1-GFP^+^ cells is illustrated in the present study by Rhodamine B isothiocyanate-dextran uptake that had leaked from the circulation in EAE animals, but was negligible in control animals (naïve, CFA). The presence of the dextran in cells in the later stages of EAE can be either due to continuous uptake because of continuously damaged BBB integrity or slow intracellular degradation while the BBB had already been repaired.

Finally, we observed the multifunctional enzyme TG2 in GFP^+^ microglial cells and monocytes/macrophages in the spinal cord lesions. Since TG2 immunoreactivity is only observed in or near lesion areas and not in ramified microglia in non-affected spinal cord, it seems that TG2 is associated with inflammatory infiltrates and/or microglial activation in mouse EAE. Interestingly, also GFP^+^ monocytes attached to the luminal side of the blood vessel were found to exhibit TG2 immunoreactivity. However, due to transcardial perfusion, these cells were scarce. Since TG2 is, amongst other functions, known to be involved in monocyte adhesion and migration in vitro (Akimov and Belkin 2001), we hypothesize that it might also be involved in the adhesion and migration processes in vivo. The presence of TG2 not only in the lesions but also at the luminal side of blood vessels supports previous findings on the involvement of TG2 in monocytes in EAE.

Furthermore, TG2 is known to contribute to apoptotic processes (Tatsukawa et al. 2016). During EAE, we observed very few cells in spinal cord lesions to express the apoptotic marker active caspase-3. This is in accordance with previous observations in mouse EAE (Irony-Tur-Sinai et al. 2009). Of the active caspase-3-immunopositive cells, single cells co-labelled with TG2 while others did not. Thus, little apoptotic processes are ongoing in EAE, and there is probably a minor role for TG2 in these processes.

Taken together, the observations in the present study showed that MOG immunization increased luminal monocyte crawling behaviour, which remained elevated during EAE. Moreover, our results revealed that circulating monocytes in EAE animals produced TG2, which may contribute to monocyte crawling and adhesion. We put forward that selective interference with monocyte crawling and migration behaviour should be applied in a very early stage of disease with the aim of counteracting disease progression. By means of the conducted experimental setup, future experiments on the effect of TG2 inhibition on monocyte behaviour can be assessed in vivo which may lead to novel treatment options for MS patients.

## Materials and methods

### Animals

**Fig. 8:**
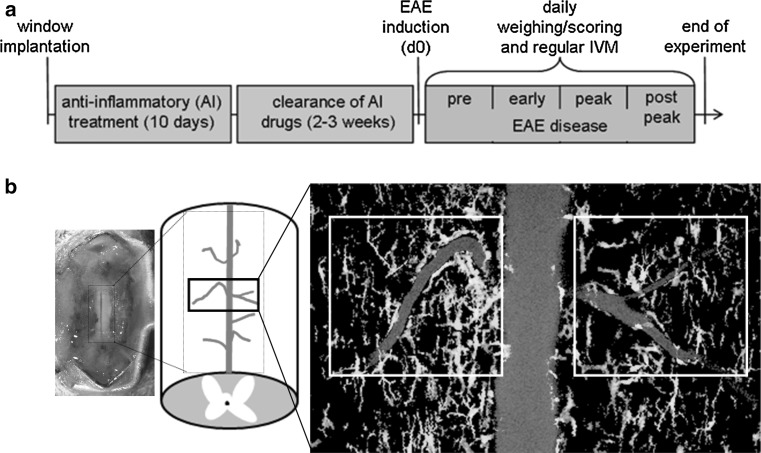
Setup of experimental procedure. **a** Spinal cord window implantation is followed by anti-inflammatory treatment and a 2- to 3-week clearance period before EAE induction and IVM; **b** IVM imaged area of the spinal cord included the venules draining into the central dorsal vein around the vertebrae level T13–L1

Adult homozygous CX3CR1^gfp/gfp^ mice, 7 weeks of age and older, were used in this study (Jung et al. 2000). All animal experiments and surgical procedures were approved by the National Animal Studies Committee of France (authorization no. 13,300), as well as approved and authorized by the National Committee for Ethic in Animal Experimentation (Section No. 14; project 86-04122012). The experimental setup of the presented study is depicted in Fig. 8a.

### Spinal cord surgery and window implantation

Microsurgical implantation of the spinal cord window was performed as described previously (Fenrich et al. 2012, 2013a). Briefly, the mouse spinal cord was exposed from the thoracic vertebra 13 (T13) to lumbar vertebra 1 (L1), and a piece of glass was fixed over the exposed spinal cord to provide permanent optical access to the exposed spinal cord (Fig. 8b). A few hours past the window implantation, the animals were highly mobile and showed little or no signs of pain. Importantly, animals with implants groomed and behaved normally, did not show any signs of motor dysfunction, and continued to gain weight. After window implantation, a layer of vascular tissue can develop between the window and CNS tissue (Fenrich et al. 2012, 2013a, b). To limit the thickness and optical density of this vascular tissue and to provide post-operative analgesia and anti-inflammatory treatment, dexamethasone (0.2 mg/kg) and Rimadyl (5 mg/kg) were injected subcutaneously every other day for 10 days.

### Induction of EAE

EAE was induced 3–4 weeks after window implantation (i.e. 2–3 weeks after cessation of analgesic and anti-inflammatory treatment) when the window was clear and no fibrosis or signs of inflammation were visible. The entire experimental scheme is shown in Fig. 8a. Mice were lightly anaesthetized with isoflurane (Baxter, 1.75% in air (v/v) for 1–2 min) to protect the spinal cord window from any strain due to mouse handling during EAE induction. Mice were injected subcutaneously in each flank and the base of the tail with a total of 75 µg myelin oligodendrocyte protein peptide (MOG_35–55_, Tocris) in complete Freund’s adjuvant (CFA), containing 800 µg *Mycobacterium tuberculosis* (BD Biosciences). In addition, mice received 400 ng pertussis toxin in PBS (Sigma Aldrich) intraperitoneally on the day of immunization and 2 days later. Animals were weighed and clinical symptoms assessed daily, as described before (Nikic et al. 2011): 0: no detectable clinical signs, 0.5: partial tail weakness, 1: tail paralysis, 1.5: gait instability and/or impaired righting ability, 2: hind limb paresis, 2.5: hind limb paresis with partial dragging, 3: hind limb paralysis, 3.5: hind limb paralysis and forelimb paresis, 4: hind limb and forelimb paralysis, 5: moribund.

### Two-photon intravital imaging

For each imaging session, mice were anaesthetized with 1.75% isoflurane for 2 min, followed by intraperitoneal injection of ketamine (100 mg/kg) and xylazine (10 mg/kg). For sessions exceeding 1 h, light anaesthesia was maintained with 0.2–0.75% isoflurane starting from about 45 min after beginning of the imaging session until completion. To acquire a visual contrast of blood vessels during imaging, mice were injected with either 2 μg of QDot-655 (Qtracker 655, non-targeted quantum dots; Invitrogen) or 2.4 mg Rhodamine B isothiocyanate-dextran 70 kDa (Sigma) in PBS, immediately before data acquisition via tail vein or retrobulbar injection. A tuneable femtosecond pulsed laser (Mai-Tai, Spectra-Physics) was used at 900 nm wavelength and coupled to an upright two-photon microscope (Zeiss, LSM 7MP) with a 20× water immersion objective lens (NA = 1.0) and five non-descanned detectors. The spinal cord window and imaged area are shown in Fig. 8b. The imaged vessels included the left and right venules draining into the central dorsal vein of the murine spinal cord. An area of 212.55 × 212.55 μm with a resolution of 0.41 μm per pixel and 5 μm distance between the individual planes of the stacks was scanned. 30–50 µm deep stacks were acquired with an acquisition rate of one plane per second. The imaging duration of the individual videos varied from 7:23 to 19:05 min and contained 35–80 stacks.

### Analysis of CX3CR1-GFP^+^ cells in the circulation

Videos and images were analysed with the ZEN lite software (Zeiss) and Fiji with the MTrackJ plug-in (Meijering et al. 2012; Schindelin et al. 2012). IVM analysis was performed on raw data, but IVM figures shown here were pseudo-coloured as well as contrast enhanced.

To analyse the behaviour of GFP^+^ cells in the blood vessels of the spinal cord, the cells were separated into two groups: (1) fast moving cells, which shortly interact with the endothelium, and (2) crawling cells that interact extensively (>25 s) with the endothelium. The presented data reflect the quantification of cells of several blood vessels from one naïve animal, one CFA animal and two EAE animals per disease stage: (1) preclinical EAE when no symptoms are apparent yet, (2) early disease, (3) peak disease and (4) post-peak disease (Fig. 8a). In addition, two mice that were asymptomatic despite MOG immunization were analysed. Per disease stage, a total of 80–340 cells present in five to ten videos were examined. Besides the number of CX3CR1-GFP^+^ cells, their intraluminal crawling movement along the endothelium was tracked and normalized to the same origin as well as medial and lateral crawling direction. All graphs were generated using GraphPad Prism 6 and microscopical image panels were created using Adobe Photoshop CS6.

### Tissue processing and immunohistochemistry

At the end of the IVM experiments, i.e. day 25–33 after EAE immunization (equivalent to post-peak disease), the mice were transcardially perfused with saline followed by 20–30 ml of 4% paraformaldehyde in 0.1 M phosphate buffer (pH 7.4). Spinal cords and spleen were dissected and post-fixed for 2 h at 4 °C. Tissue was cryo-protected with 10% sucrose in PBS and embedded in OCT tissue tek (VWR Chemicals). Coronal sections (10 μm) were cut, dehydrated and stored at −80 °C until immunostaining.

For single immunohistochemical stainings, sections were thawed, blocked in 3% BSA in TBS containing 0.5% Triton X-100 (TBS-T; Sigma), and incubated with primary antibodies for Iba1, CD68 (rat), CD3 or CD45 (Table 1). For double staining of CD45 and CD68 (rabbit), incubation of both primary antibodies was done at once. All sections were incubated with primary antibodies at 4 °C overnight followed by three 5 min washes in PBS. The sections were then incubated with appropriate AF594 or AF633-labelled IgGs (1/400, Molecular probes) in TBS-T for 2 h at room temperature.

**Table 1:** Primary antibodies for immunohistochemistry

Target	Origin	Dilution	Supplier	Code
Iba1	Rabbit	1/1000	Wako chemicals	019-19741
CD68	Rat	1/300	Serotec	MCA1957
CD68	Rabbit	1/400	Abcam	ab125212
CD45	Rat	1/10	Gift from dept. Molecular Cell Biology and Immunology, VUmc, Amsterdam	clone MP33
CD3	Rat	1/100	AbD Serotec	MCA500G
NKp46-biotin	Goat	1/100	R&D Systems	BAF2225
TG2	Mouse	1/100	Prof T. J. Johnson Sheffield University, UK (gift)	Clone IA-12
Cleaved caspase-3 (Asp175); 17/19 kDa fragments	Rabbit	1/350	Cell Signaling Technology	#9661

Before staining of NKp46 (Table 1), endogenous peroxidases were quenched (0.3% H_2_O_2_, 0.1% sodium azide in TBS) and sections incubated with primary antibody for 1.5 h at room temperature. After washing, ImmPRESS HRP anti-goat IgG polymer detection kit (Vector laboratories) was added for 30 min before slides were washed and incubated for 10 min with the TSA™ reagent Alexa Fluor 594^®^ tyramide (1/100 in amplification buffer, Thermo Fisher Scientific).

Immunohistochemical co-labelling of TG2 and the above used immune cell markers (Table 1) required pre-treatment with a mouse on a mouse blocking kit (M.O.M.; Vector Laboratories) according to the manufacturer’s guidelines. Briefly, the sections were blocked for 1 h with blocking reagent and the staining was continued as described above with additional AF633-labelled donkey anti-mouse IgG’s (1/400, Molecular probes) to detect TG2.

For double-labelling of TG2 and cleaved caspase-3 fragments (17/19 kDa) (Table 1), endogenous peroxidases were blocked, M.O.M blocking was applied and sections were incubated with antibodies as described above. ImmPRESS AP anti-rabbit IgG polymer detection kit (Vector laboratories) was added for 30 min, slides were washed and EnVision+ kit HRP anti-mouse (Dako) was applied for 30 min. After washing, the slides were first incubated with liquid permanent red (LPR, Dako) as AP substrate and then after a quick wash with DAB+ (Dako) as HRP substrate, both according to manufacturer guidelines.

All slides were coverslipped with polyvinyl alcohol mounting medium with DABCO (Fluka) and all fluorescent slides were examined with a confocal laser-scanning microscope or a fluorescence microscope (Leica TSC-SP2-AOBS and Leica DM5000B, Leica Microsystems). The light-microscopical double staining for cleaved caspase-3 fragments (17/19 kDa) and TG2 was analysed with a Nuance spectral imaging camera (Nuance) and displayed in colours representing fluorescent staining to increase the visual contrast. As negative staining control, primary antibodies were omitted, which resulted in minor or no background immunoreactivity (data not shown).

### Statistical analysis

IVM data were not normally distributed and therefore statistically analysed using the non-parametric Kruskal–Wallis test followed by Dunn’s multiple comparison test using GraphPad Prism 6. Differences were considered significant if *p* < 0.05, and considered a trend if *p* < 0.06.

## Electronic supplementary material

Below is the link to the electronic supplementary material.

Supplementary material 1 (TIFF 539 kb)

Supplementary material 2 (TIFF 448 kb)

Supplementary material 3 (DOCX 15 kb)

## Abbreviations

CFA: Complete Freund’s adjuvant
CNS: Central nervous system
EAE: Experimental autoimmune encephalomyelitis
GFP: Green fluorescent protein
IVM: Intravital microscopy
MOG: Myelin oligodendrocyte glycoprotein
MRI: Magnetic resonance imaging
MS: Multiple sclerosis
TG2: Tissue transglutaminase
