# Supplementary figures and images for: Monocyte behaviour and tissue transglutaminase expression during experimental autoimmune encephalomyelitis in transgenic CX3CR1gfp/gfp mice
Source: Amino Acids. 2016 Nov 9;49(3):643–58. doi: 10.1007/s00726-016-2359-0 (PMC5332504; doi:10.1007/s00726-016-2359-0)

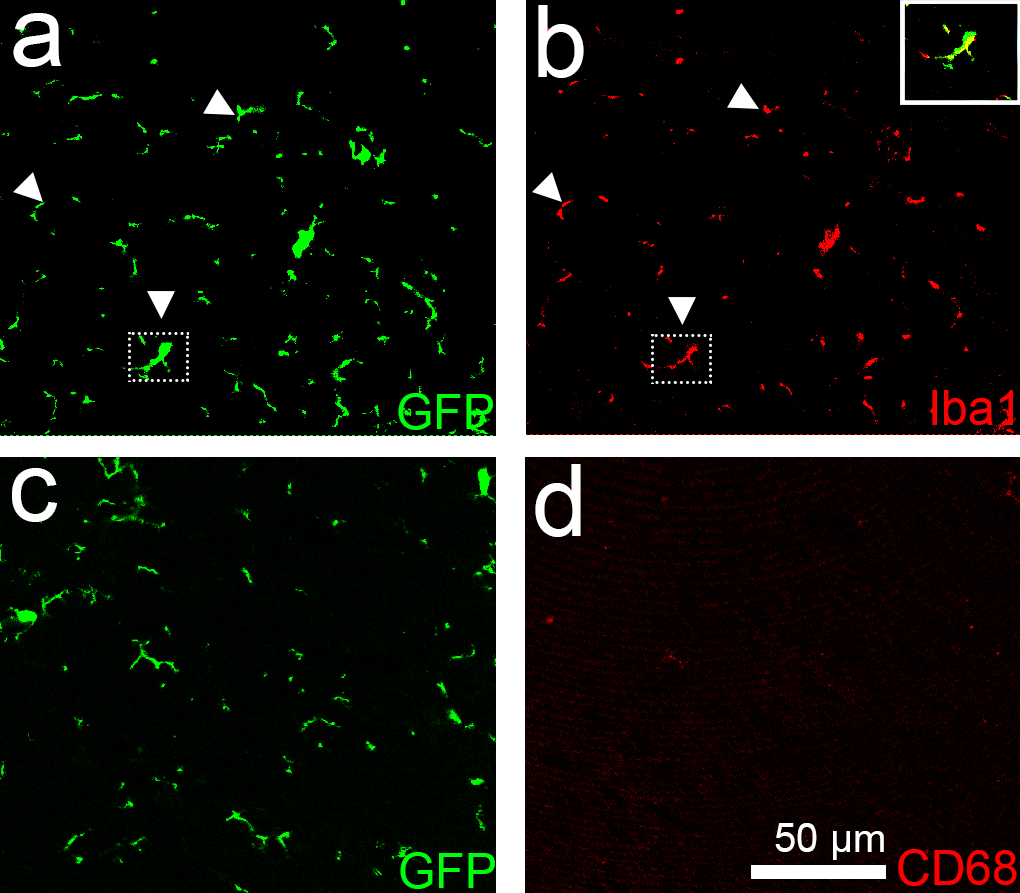

Supplement: Supplementary file 1 — Supplementary material 1 (TIFF 539 kb) [file 726_2016_2359_MOESM1_ESM.tif]

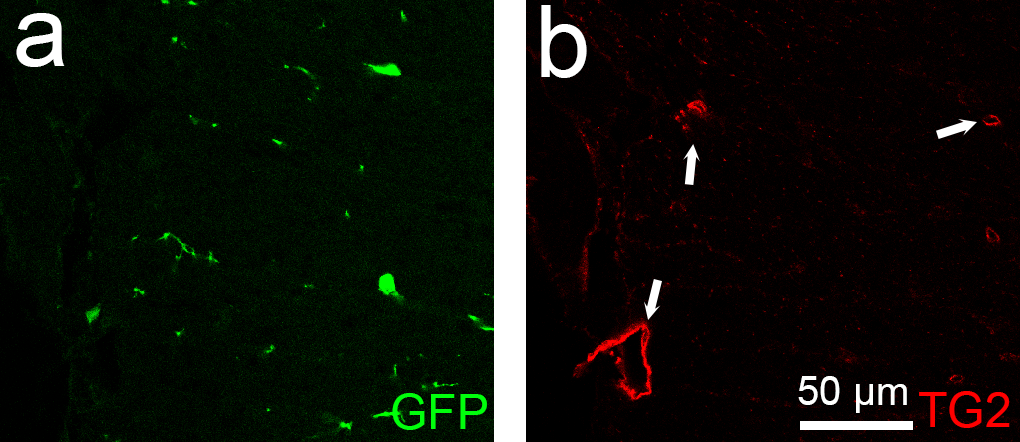

Supplement: Supplementary file 2 — Supplementary material 2 (TIFF 448 kb) [file 726_2016_2359_MOESM2_ESM.tif]
