# Supplementary material for: Monocyte behaviour and tissue transglutaminase expression during experimental autoimmune encephalomyelitis in transgenic CX3CR1gfp/gfp mice
Source: Amino Acids. 2016 Nov 9;49(3):643–58. doi: 10.1007/s00726-016-2359-0 (PMC5332504; doi:10.1007/s00726-016-2359-0)
Supplement: Supplementary file 3 — Supplementary material 3 (DOCX 15 kb) [file 726_2016_2359_MOESM3_ESM.docx]

**Supplementary data**

**Disease-course dependent changes in monocyte behaviour during experimental multiple sclerosis in transgenic CX3CR1^gfp/gfp^ mice**

Navina L. Chrobok, Alexandre Jaouen, Keith K. Fenrich, John G. J. M. Bol, Micha M. M. Wilhelmus, Benjamin Drukarch, Franck Debarbieux and Anne-Marie van Dam

**Legends**

**Supplementary Figure 1**

**(a,c)** CX3CR1-GFP^+^ cells in the spinal cord tissue of a CFA induced control animal (n=1) exhibit ramified microglial like morphology. These cells show immunoreactivity for **(b)** Iba1 but not **(d)** CD68, indicating a resting microglial phenotype. Arrowheads: co-labelled cells (higher magnification in the insert).

**Supplementary Figure 2**

**(a)** CX3CR1-GFP^+^ cells in the spinal cord tissue of a CFA induced control animal (n=1) exhibit no immunoreactivity for **(b)** TG2. TG2 immunoreactivity is only found in blood vessels (arrows).
